# A New Method for Evaluating Pelvic and Trunk Rotational Pitching Mechanics: From Qualitative to Quantitative Approaches

**DOI:** 10.3390/ijerph18030905

**Published:** 2021-01-21

**Authors:** Yu-Chuan Lin, Paul Pei-Hsi Chou, Hwai-Ting Lin, Chia-Lung Shih, Cheng-Chang Lu, Fong-Chin Su

**Affiliations:** 1 Department of Biomedical Engineering, National Cheng Kung University, Tainan 70101, Taiwan; 910095@ms.kmuh.org.tw; 2 Division of Sports Medicine, Department of Orthopaedic Surgery, Kaohsiung Medical University Hospital, Kaohsiung 80708, Taiwan; arthroscopy.pc@gmail.com (P.P.-H.C.); cclu0880330@gmail.com (C.-C.L.); 3 Department of Orthopaedics, School of Medicine, College of Medicine, Kaohsiung Medical University, Kaohsiung 80708, Taiwan; 4 Department of Sports Medicine, College of Medicine, Kaohsiung Medical University, Kaohsiung 80708, Taiwan; whiting@kmu.edu.tw; 5 Department of Medical Research, Ditmanson Medical Foundation Chiayi Christian Hospital, Chiayi 60002, Taiwan; stone770116@gmail.com; 6 Department of Orthopaedic Surgery, Kaohsiung Municipal Hsiao-Kang Hospital, Kaohsiung 81267, Taiwan

**Keywords:** baseball, pelvis, trunk, kinematics, performance

## Abstract

The purpose of this study was to build on existing qualitative to quantitative approaches to develop a new quantitative method for evaluating pelvic and trunk rotational pitching mechanics. Thirty pitchers were divided into two groups (“Pattern1”: closed “hip-to-shoulder separation”; “Pattern2”: open “hip-to-shoulder separation”). Several parameters were analyzed. Higher ball speeds were found in group of Pattern1, four key characteristics of which were identified. Based on the results, a new evaluation method was developed. Pelvic and trunk rotational mechanics were classified into four types. Type1 (proper mechanics) enabled significantly higher ball speed than the other three types and was thought to involve proper energy transfer from the stride foot to the throwing upper limb. Types 2–4, however, were regarded as “improper mechanics”, which could result in slower ball speeds and less efficient energy transfer. A qualitative approach, based on “expert opinion”, can specify optimal pelvis and trunk rotational mechanics. However, quantitative analysis is more precise in identifying three improper types of pelvis and trunk rotational mechanics. Furthermore, special programs, such as core strengthening and flexibility training, can be developed for various improper practices in order to improve pitching mechanics.

## 1. Introduction

Pitching mechanics make up part of a kinetic chain in which energy or momentum is transferred from the striding foot through to the throwing hand [1,2]. Suboptimal pitching mechanics are thought to diminish parameters of performance such as ball speed and to increase the risk of injuries such as overload in joints [3,4]. Various studies have focused on improper pitching mechanics in the extremities [5,6,7,8]. Pelvis and trunk, which are also described as “runners in this relay race of energy transfer”, play important roles. The literature features two common approaches to describing pelvic and trunk rotational mechanics. The qualitative approach is to identify certain features such as “early trunk rotation” [9] and “hip-to-shoulder separation” [5,10,11,12]. The quantitative method draws on kinematic data regarding the pelvis and trunk, such as the pelvic/trunk rotation angle, pelvic/trunk orientation, trunk separation, and spinal rotation [13,14,15,16,17,18].

Although there are various descriptions in the literature, there have yet to be any studies that approach pelvic and trunk rotational mechanics in highly systematic way. First, we seek to rectify that by starting from a qualitative perspective. Hip-to-shoulder separation refers to the position of the hips relative to the shoulder just prior to foot contact being made. It is a commonly-employed qualitative method and is described in detail by Erickson et al. [10]. We compared the parameters that differentiated the closed and open hip-to-shoulder separation patterns and hypothesized that a closed pattern produced a higher ball speed. Moreover, we aimed to determine the parameters that significantly differed between these two patterns. These parameters can be further utilized for quantitative analysis. Second, pelvis and trunk are linkage parts of axial body structure. In most of the quantitative kinematic studies in the literature [13,14,15,16,17,18], features of pelvic and trunk rotation were analyzed separately. We thought it might be useful to understand more about the characteristics of pelvic and trunk rotational mechanics by placing the kinematic curves of the pelvis and trunk together. Finally, the aim of these procedures was to develop a new quantitative method, based on the results of quantitative analysis, which constituted a more precise way to evaluate pelvic and trunk rotational pitching mechanics.

## 2. Materials and Methods

### 2.1. Participants

Thirty adult male elite pitchers were enrolled for this study. All of them were right-dominant handed and without a history of surgeries due to injuries sustained while pitching. Informed consent documents, which were approved by the Institutional Review Board of the affiliated institutions (KMUHIRB-SV(I)-20180022), were signed by all of the participants.

### 2.2. Procedures

This study was conducted outdoors, in a baseball stadium. Following stretching and warm-up, each participant threw ten overhand fastballs with maximum effort from the pitching mound toward a catcher at the home plate. Protocols of the warm-up, markers placement and experimental setup were similar to those cited in our previous studies [19,20,21]. For each pitching task, reflective markers were attached to the participants and tracked by a motion capture system (Motion Analysis Corporation, Santa Rosa, CA, USA) that comprised eight charge-coupled device cameras with a sampling frequency of 300 Hz. The ball speed in each pitch was measured using a radar gun (Jugs Sports International Distributors, Tualatin, OR, USA). The position of the tracked markers was then used for the estimation of joint centers, three-dimensional body-segment locations, and kinematics during each pitching task.

**Figure 1:**
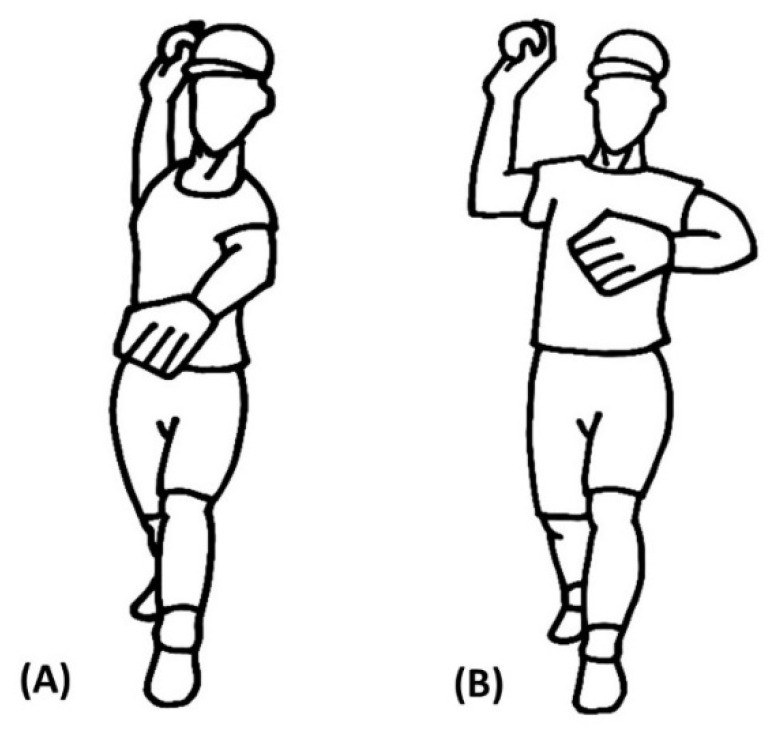
(**A**) Closed pattern of “hip-to-shoulder separation”; (**B**) Open pattern of “hip-to-shoulder separation”.

All pitches were divided into two patterns by one expert (a pitching coach from a professional team) in accordance with a closed and open pattern of hip-to-shoulder separation [10]. The expert watched videos of each pitch from near the catcher’s viewpoint. At the moment of foot contact, pitches in which a complete “arm–elbow” structure of the throwing arm was not visible were identified as a closed pattern of hip-to-shoulder separation (Figure 1A). At the moment of foot contact, pitches in which a complete arm–elbow structure of the throwing arm was visible were identified as conforming to the open pattern of hip-to-shoulder separation (Figure 1B). Pitches that were ambiguous were excluded. The participants who had more than five pitches of closed pattern were assigned to the group of Pattern1. The participants who had more than five pitches of open pattern were assigned to the group of Pattern2. For each participant in Pattern1, his top five fastest closed pattern pitches were selected for analysis. Similarly, the top five fastest open pattern pitches were selected for analysis from the group of Pattern2. Ultimately, 30 participants with 150 fastballs were enrolled. Demographics of the two groups is presented in Table 1.

**Figure 2:**
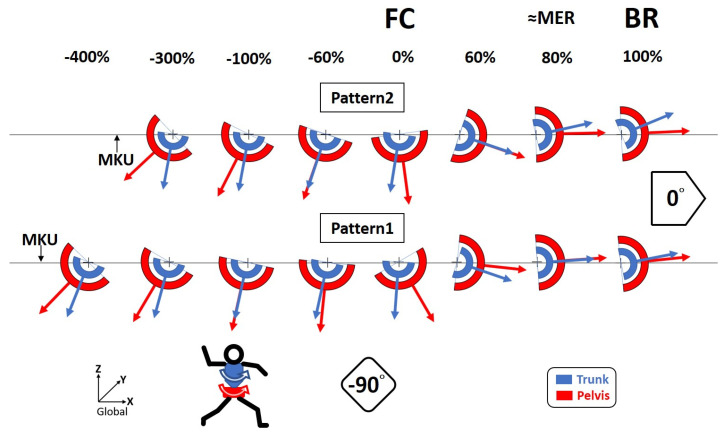
A schematic diagram comparing Pattern1 and Pattern2 viewing in the transverse plane at different time ratios. The kinematic definition of pelvic and trunk axial rotation is in relation to global coordinate system. In the transverse plane, rotation towards the home plate is defined as 0°, whereas that towards third base is defined as −90°. MKU = maximum-knee-up; FC = foot contact; MER = maximum -shoulder-external-rotation; BR = ball-release.

The kinematic definition of pelvic and trunk axial rotation is in relation to global coordinate system. In the transverse plane, rotation towards the home plate is defined as 0°, whereas that towards third base is defined as −90° (Figure 2).

### 2.3. Parameters

The parameters derived from the kinematic data (Table 2) were used in the approach, including: (1) parameters of the timing of events; (2) parameters of the angle at the time of the events; (3) parameters of trunk–pelvis separation (TPS) at the time of the events; (4) parameters associated with special time events and intervals; (5) parameters that are important in the stride phase; and (6) the ball speed.

#### 2.3.1. Parameters on the Timing of Events

There are five hallmark timing events associated with a baseball pitch, including maximum-knee-up (MKU), foot contact (FC), maximum shoulder external rotation (MER), ball-release (BR) and maximum shoulder internal rotation (MIR) [6]. The time interval between FC and BR, abbreviated as “BRt-FCt”, was used for the normalization. The time of the FC was set as 0, and that of BR as 100%. The “time ratio” was calculated for the normalization timing of events between pitches. The formula of the time ratio of the event is as shown below:

Time ratio (%) of the event = (event time − FC time) × 100/(BR time − FC time)

For example:

The time ratio of MKU (MKUr) = (MKU time − FC time) × 100/(BR time − FC time)

“MKUr” is the abbreviated form of the “time ratio of MKU”, “MERr” is the abbreviated form of the “time ratio of MER”, and “MIRr” is the abbreviated form of the “time ratio of MIR”. The parameters of the timing of events include: “BRt-FCt”, “MKUr”, “MERr” and “MIRr”.

#### 2.3.2. Parameters of the Angle at the Time of Events

“TAoMKU” is the abbreviated form of “trunk angle at the moment of MKU”, “PAoMKU” is the abbreviated form of “pelvic angle at the moment of MKU”, and so on. The parameters of the angle at the time of events include: “TAoMKU”, “TAoFC”, “TAoMER”, “TAoBR”, “TAoMIR”, “PAoMKU”, “PAoFC”, “PAoMER”, “PAoBR” and “PAoMIR”.

#### 2.3.3. Parameters of Trunk–Pelvis Separation at the Time of Events

Trunk–pelvis separation (TPS) is defined as trunk angle minus the pelvic angle at the same moment. The TPS is similar to the trunk axial rotation angle related to pelvic co-ordinates, and in the literature, the value of TPS is equivalent to “trunk separation”, “trunk twist” and “spinal rotation” in the literature [14,16,17,18]. “TPSoMKU” is the abbreviated form of “TPS at the moment of MKU”, etc. The parameters of the TPS at the time of events include: “TPSoMKU”, “TPSoFC”, “TPSoMER”, “TPSoBR”, and “TPSoMIR”.

#### 2.3.4. Parameters Associated with Special Time Events and Intervals

**Figure 3:**
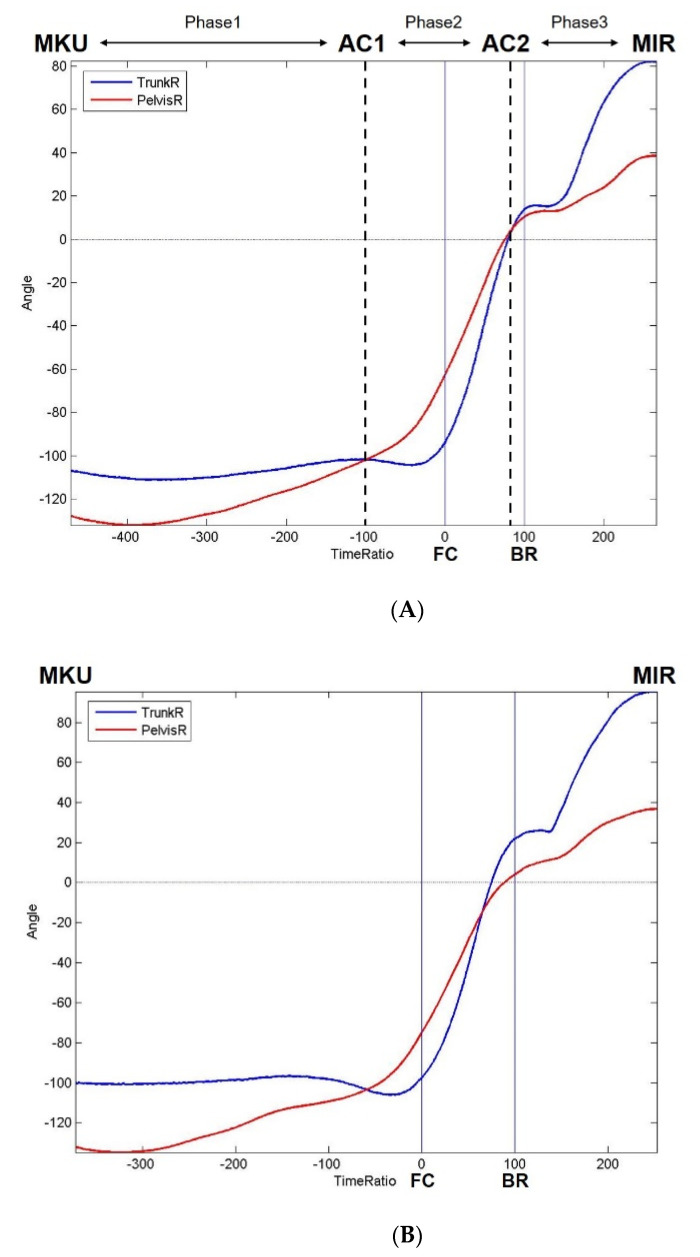
(**A**) Curves of mean rotational angle of pelvis and trunk related to time ratio from MKU to MIR of Pattern1. It shows two special time events: AC1 and AC2, as well as three novel phases. (**B**) Curves of mean rotational angle of pelvis and trunk related to time ratio from MKU to MIR of Pattern2. AC1 = time of first pelvic and trunk angle crossing; AC2 = time of second pelvic and trunk angle crossing; BR = ball-release; FC = foot contact; MIR = maximum -shoulder-internal-rotation; MKU = maximum-knee-up.

Two special time events are acknowledged when plotting the curves of pelvic angle and trunk angle together (Figure 3A).

One event is the first crossing of curves of the pelvic and trunk angles (the first angle crossing is abbreviated as “AC1”). Another event is the second crossing of the curves of the pelvic and trunk angles (the second angle crossing, abbreviated as “AC2”). “AC1r” is the abbreviated form of the “time ratio of AC1”. “AC2r” is the abbreviated form of the “time ratio of AC2”. “AC2r-AC1r” is the abbreviated form of the “time ratio interval between AC1 and AC2”. The parameters associated with special time events & intervals include, “AC1r”, “AC2r” and “AC2r-AC1r”.

#### 2.3.5. Parameters That Are Important during the Stride Phase

Several studies have noted that some parameters during the stride phase, such as stride length, maximum knee height and stride foot contact direction, are important and may affect the ball speed or some of the pitching mechanics [4,22]. “StrideL/BH” is the abbreviated form of the “percentage of stride length normalized with body height”. “MKH/BH” is the abbreviated form of the “percentage of maximal knee height normalized with body height”. “SFCD” is the abbreviated form of “stride foot contact direction”. The definition of “SFCD” is the same as the kinematic definition of this study. In the transverse plane, the SFCD towards home plate is defined as 0°, whereas that towards third base is defined as −90°. The parameters that are important in the stride phase include, “StrideL/BH”, “MKH/BH” and “SFCD”. Finally, the resulting parameter is “Ball Speed”.

### 2.4. Statistical Analyses

Statistical analyses were conducted using SPSS 12.0 (SPSS Inc., Chicago, IL, USA) software. The mean data from five pitches by each subject were used for the analysis. The independent t test was used for comparison of each of the parameters between the two groups. A statistically-significant α level was set a priori to 0.05. Cohen’s *d* effect sizes were calculated. Then, the Pearson *r* correlation between each parameter and the ball speed was calculated. For multiple comparisons, the false discovery rate (FDR)-adjusted p-values were calculated using the R package “qvalue”. If a parameter with an FDR-adjusted p-value was smaller than 0.05, this was related to ball speed and adopted for further analysis. A receiver operating characteristic (ROC) curve analysis was employed to determine the best cut-off value of these parameters in order to discriminate between low and high ball speeds.

## 3. Results

A schematic diagram comparing Pattern1 and Pattern2 viewing in the transverse plane at different time ratios is presented in Figure 2. The curves of mean rotational angle of pelvis and trunk related to time ratio from MKU to MIR of Pattern1 and Pattern2 are shown in Figure 3A,B.

**Figure 4:**
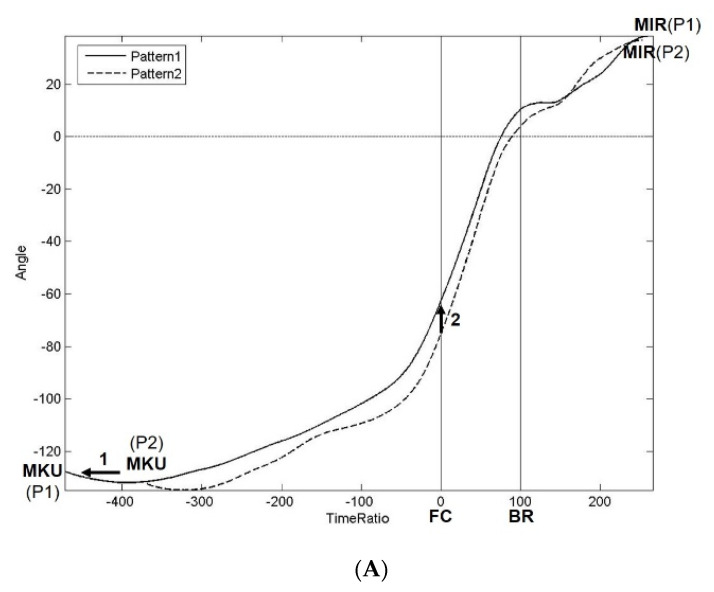

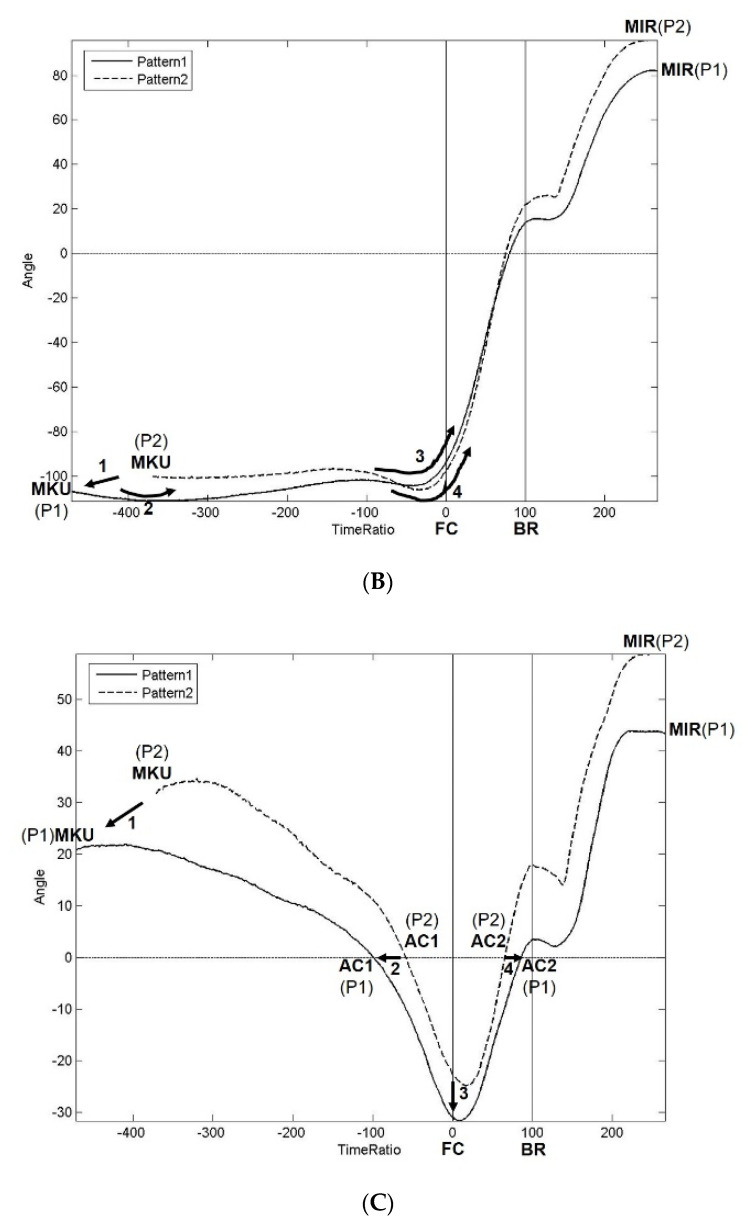
(**A**) Curves of mean pelvic angle of Pattern1 and Pattern2 related to time ratio from MKU to MIR. Arrow1 indicates that Pattern1 has an earlier MKU than Pattern2. Arrow2 indicates that Pattern1 has more of a leading pelvic rotational angle at the moment of foot contact (“PAoFC”) than Pattern2. (**B**) Curves of mean trunk angle of Pattern1 and Pattern2 related to time ratio from MKU to MIR. Arrow1 indicates that Pattern1 has an earlier MKU than Pattern2. Pattern1 entails the backward rotation of the trunk in Phase1 (Arrow2), whereas Pattern2 does not feature this behavior. Pattern2 shows a turning-back of the trunk prior to foot contact in Phase2 (Arrow4), whereas Pattern1 does not present this tendency (Arrow3). (**C**) Curves of mean trunk–pelvis separation (TPS) of Pattern1 and Pattern2 related to time ratio from MKU to MIR. Arrow1 indicates that Pattern1 has an earlier MKU and less of a TPS in Phase1 due to the backward rotation of the trunk. Pattern1 has an earlier AC1 (Arrow2) and later AC2 (Arrow4) than Pattern2. Pattern1 exhibits more of an absolute value of negative TPS at the moment of foot contact (“TPSoFC”) than Pattern2 (Arrow3). Note that Pattern1 and Pattern2 have different mean time ratios of MKU, MIR, AC1 and AC2 in (**A**–**C**). AC1 = time of first pelvic and trunk angle crossing; AC2 = time of second pelvic and trunk angle crossing; BR = ball-release; FC = foot contact; MIR = maximum-shoulder-internal-rotation; MKU = maximum-knee-up; P1 in parentheses = Pattern1; P2 in parentheses = Pattern2.

The curves of mean pelvic angle of Pattern1 and Pattern2 related to time ratio from MKU to MIR and those of trunk are shown in Figure 4A,B. The curves of mean TPS of Pattern1 and Pattern2 related to time ratio from MKU to MIR are shown in Figure 4C. It can be noted that Pattern1 and Pattern2 have different mean time ratios of MKU, MIR, AC1, and AC2.

The results of the parameters of the timing of events are shown in Table 3. The “MKUr” of Pattern1 is significantly earlier than that of Pattern2.

The results of the parameters of the angle at time events are shown in Table 3. The “TAoMKU”, “TAoMER”, “TAoBR” and “TAoMIR” of Pattern1 significantly trail behind Pattern2. However, the “PAoFC”, “PAoMER” and “PAoBR” of Pattern1 are leading.

The results of the parameters of the TPS at time events are shown in Table 3. All of the parameters listed here significantly differ between Pattern1 and Pattern2.

The results of the parameters associated with special time events and intervals are shown in Table 3. The “AC1r” of Pattern1 is significantly earlier than in Pattern2. In contrast, the “AC2r” of Pattern1 is later than in the case of Pattern2. Additionally, the intervals between AC1 and AC2 (“AC2r–AC1r”) are significantly longer in Pattern1 than in Pattern2.

The results of parameters that are important in stride phase are displayed in Table 3. No significant differences were identified between Pattern1 and Pattern2. The results of the ball speed are shown in Table 3. The “Ball Speed” was significantly higher in Pattern1 than in Pattern2.

Table 4 presents the correlation coefficients and cut-off values from the ROC of each parameter with statistically-significant correlations with ball speed. Cohen’s *d* effect sizes of these parameters are also shown in Table 4. The parameter with the highest correlation with ball speed is “PAoFC”. It also has the largest Cohen’s *d* effect size. The cut-off value of “PAoFC” from the ROC can be used as a reference to discriminate pitches between low ball speed (with “PAoFC” < −69.95°) and high ball speed (with “PAoFC” > −69.95°). Other parameters with statistically-significant correlations with the ball speed are listed in Table 4.

## 4. Discussion

The rotation of the pelvis and trunk is an area that merits analysis and may be key factors in pitching mechanics. Therefore, it is important to understand and follow a scientific method to evaluate these mechanics. This study seeks to systematically incorporate both qualitative and quantitative approaches to this. The goal of the former is to identify features between different patterns of “hip-to-shoulder separation”. The novelty of this study is placing the curves of the pelvis and trunk together. By doing so, the characteristics of pelvic and trunk rotational mechanics can be more clearly seen. Three phases of pelvic and trunk rotation during pitching are introduced herein. Closed pattern of “hip-to-shoulder separation” was found to have a higher ball speed. Four characteristics of pelvic and trunk rotation of pitches with closed “hip-to-shoulder separation” were also identified. These lend fresh perspective to pitching mechanics.

### 4.1. Three Novel Phases of Pelvic and Trunk Rotation during Pitching

As was noted above, there were two special time events, the first angle crossing (AC1) and the second angle crossing (AC2), which were noted while plotting the curves of the pelvic and trunk angles together. We divided a pitching cycle involving the pelvis and trunk into three distinct phases based on these time events (Figure 3A). The definition of Phase1 is the interval from MKU to AC1. In Phase1, the angle of the pelvis is more backward behind the trunk, and so the TPS is positive. The definition of Phase2 is the interval from AC1 to AC2. In Phase2, the angle of the pelvis goes beyond the trunk, and the TPS becomes negative. The definition of Phase3 is the interval from AC2 to MIR. In Phase3, the trunk rotates over the pelvis (positive TPS) again.

The results show that Pattern1 has a significantly faster ball speed than Pattern2, which implies that Pattern1 has better rotational mechanics of the pelvis and trunk for faster ball speeds. We will discuss and explain the characteristics of Pattern1 according to the stated three phases of pelvic and trunk rotation during pitching.

#### 4.1.1. Phase1

The pelvis functions like a rocket booster, and the trunk, proceeding with the metaphor, is the spacecraft. In Phase1, the goal is pre-tension of the pelvis for elastic energy storage, followed by pelvic run-up for the preparation of boosting. The MKU is the initial moment of the stride phase and can be thought of as the timing of the start-up of the pelvic backward rotation for elastic energy storage [22]. In plots of the pelvic rotational angle, the trunk rotational angle and TPS, Pattern1 has an earlier MKU (“MKUr”) than Pattern2 (Figure 4A, Arrow1; Figure 4B, Arrow1; Figure 4C, Arrow1). In addition, Pattern1 exhibits backward rotation of the trunk following the pelvis (Figure 4B, Arrow2), whereas Pattern2 does not feature this behavior. The backward rotation of the trunk results in a decrease in the maximum TPS in Phase1 (Figure 4C, Arrow1). In summary, during Phase1, Pattern1 shows characteristics with the curve of the pelvic rotational angle being shifted earlier (Figure 4A) and that of the trunk rotational angle being pressed down (Figure 4B).

#### 4.1.2. Phase2

In Phase2, the goal is to boost the pelvis first, followed by an acceleration of the trunk. In the plot of the TPS, Pattern1 had an earlier AC1 (“AC1r”) and later AC2 (“AC2r”) than Pattern2 (Figure 4C Arrow2, Arrow4). In other words, Pattern1 represented a longer period of Phase2 (“AC2r-AC1r”). Luera et al. studied the kinematic characteristics of high school pitchers versus professionals [13] and found that high school pitchers were incapable of rotating their trunks and pelvises to aid in pitching. Therefore, high school pitchers primarily threw hard by generating larger forces in their elbows and shoulders, which may increase their risk of injury. In the study, the figure on upper trunk rotation was similar to the plot of the TPS in this work. This was because definition of trunk rotation in their study was in relation to pelvic coordinate system, rather than global one. The time period between the first and second zero-crossing of the mean upper trunk rotational angle was longer in the group of professional pitchers, who had a correspondingly faster ball speed. This accords with the result of our study that Pattern1 (with faster ball speeds) has a longer period of Phase2 (“AC2r-AC1r”).

In the plot of the pelvic rotational angle, Pattern1 demonstrates more leading pelvic rotational angle at the moment of FC (“PAoFC”) than Pattern2 does (Figure 4A, Arrow2). Oi et al. compared the difference between Japanese and American pitchers [15]. American pitchers threw with a higher ball velocity than their Japanese counterparts. The American group exhibited more leading pelvic rotation angle at the instant of lead foot contact than the Japanese one. Wright et al. noted that pitchers who were defined as “early pelvis rotators” (more leading “PAoFC”) displayed greater shoulder external rotation at the moment of FC and the earlier occurrence of maximal pelvic rotation angular velocity [23].

In the plot of the TPS, Pattern1 shows more of an absolute value of the negative TPS (trunk trailing behind the pelvis) at the moment of FC (“TPSoFC”) than Pattern2 (Figure 4C, Arrow3). This result was also reported in the study by Luera et al., with the finding that professional pitchers with a higher pitch velocity had significantly greater upper trunk rotation (equivalent to “TPSoFC”) [13]. Nissen et al. noted that the relative difference in rotation between the pelvis and trunk at FC (equivalent to “TPSoFC”) was 28° with greater external rotation of the trunk in relation to pelvis [17]. They assumed that this difference in rotation enabled “coiling” whereby potential energy was built up and subsequently transferred to the arm. Fleisig et al. observed the biomechanical changes in youth pitchers between the ages of nine to 15. They noted that trunk separation and ball velocity both increased with age [16].

In the plot of the trunk rotational angle, Pattern2 features a turning-back of trunk prior to FC (Figure 4B, Arrow4), whereas Pattern1 does not exhibit this behavior (Figure 4B, Arrow3). In summary, during Phase2, Pattern1 shows characteristics in terms of the curve of the pelvic rotational angle being pulled up far from the curve of the trunk rotational angle (Figure 4A). This results in the expanding and shifting down of the entire TPS curve (Figure 4C). Moreover, Pattern1 displays characteristics with no turning-back of the trunk rotational angle prior to the FC (Figure 4B).

#### 4.1.3. Phase3

In Phase3, the goal is slowing down the pelvis and trunk to achieve a sudden “stop” for a relatively stable condition. In this condition, energy can be more efficiently transferred to the throwing arm. This was consistent with a study by Dun et al. [14], who noted that energy can be transferred in a more effective way if the lower body segment was stabilized while the upper one was in movement or rotation. After the ball has been released, which means the task is complete, the pelvis and trunk continue to rotate forward for the follow-through and unloading, decreasing the risk of injury.

### 4.2. Characteristics of Pattern1 (Closed Hip-to-Shoulder Separation)

In summary, the Pattern1 pitchers display several characteristics (described as below and illustrated in Figure 4A–C) that distinguish them from their counterparts in Pattern2, with slower ball speed. These are as follows:

(1) They rotate their trunks backwards following the pelvises in Phase1 (Figure 4B).
(2) They commence rotation of their pelvises (backwards and then forwards) earlier in Phase1 (Figure 4A).
(3) They achieve a more leading pelvic angle (Figure 4A) and gain more angle between the pelvis and trunk around the moment of foot contact in Phase2 (Figure 4C).
(4) They do not rotate their trunks backwards in Phase2 just before foot contact while pitchers in Pattern2 rotate their trunks backwards (Figure 4B).

These four characteristics may be critical to proper pelvic and trunk rotational mechanics for faster ball speeds. They can be taken as references to help pitchers and coaches evaluate and improve their pitching mechanics.

### 4.3. Evaluation of Pelvic and Trunk Rotational Mechanics Using “PAoFC” Accompanied by “TPSoFC”

**Figure 5:**
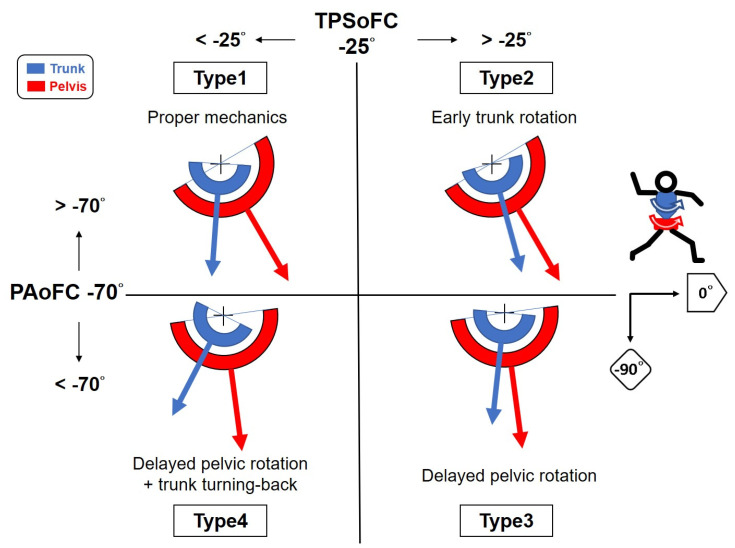
Four types of pelvic and trunk rotational pitching mechanics (viewing in the transverse plane). A “PAoFC” (Pelvic angle at the moment of foot contact) of −70° and “TPSoFC” (Trunk-pelvis separation at the moment of foot contact) of −25° were used for classification based on the result of the cut-off values from ROC (receiver operating characteristic). Type1 (proper mechanics) represents a leading “PAoFC” and is followed by the trunk with enough separation between the pelvis and trunk. Type2 (early trunk rotation) represents a leading “PAoFC” and is followed by the trunk with insufficient separation between the pelvis and trunk. Type3 (delayed pelvic rotation) represents a fallen behind “PAoFC” and is followed by the trunk with insufficient separation between the pelvis and trunk. Type4 (delayed pelvic rotation with trunk turning-back) represents a fallen behind “PAoFC” and is followed by the trunk with enough separation between the pelvis and trunk. Typically, the adequate separation of Type4 results from a turning-back of trunk rotation prior to foot contact. Types 2–4 are regarded as being characterized by “improper mechanics”.

For sports scientists or coaches/pitchers who wish to more precisely realise pitching mechanics, the recognition of closed and open ‘hip-to-shoulder separation’ by experts is insufficient. In accordance with the results of and characteristics displayed by Pattern1 in this study, we develop a more objective method for evaluating pelvic and trunk rotational pitching mechanics. “PAoFC”, which is the parameter with the highest correlation with ball speed and the largest effect size, is used as the primary component of this method, accompanied by “TPSoFC”. A “PAoFC” of −70° and “TPSoFC” of −25° are used here for classification based on the result of the cut-off values from the ROC. Thus, pitches can be classified in terms of four types of pelvic and trunk rotational mechanics (Figure 5). Type1 represents a leading pelvic rotational angle at the moment of FC and is followed by the trunk with enough separation between the pelvis and trunk. This is regarded as “proper mechanics”. Type2 represents a leading pelvic rotational angle at the moment of FC and is followed by the trunk, but with insufficient separation between the pelvis and trunk. This is considered as “early trunk rotation”. Type3 represents a pelvic rotational angle that falls behind at the moment of FC and is followed by the trunk with insufficient separation between the pelvis and trunk. This is referred to as “delayed pelvic rotation”. Type4 constitutes a pelvic rotational angle that falls behind at the moment of FC and is followed by the trunk with enough separation between the pelvis and trunk. Typically, the separation of Type4 results from the turning-back of the trunk’s rotation prior to FC. Pitchers who feature Type4 mechanics may attempt to increase the angle between the pelvis and trunk by backward trunk rotation in order to gain more “coiling” for potential energy. This is defined as “delayed pelvic rotation with trunk turning-back”. Types 2, 3, and 4 are all regarded as “improper mechanics”. As is shown in Table 5, Type1 (proper mechanics) features significantly higher ball speed than the other three types, whereas Types 2–4 (improper mechanics) exhibit no significant difference in ball speed between one another.

For the “expert decision”, as is shown in Table 5, 53 of 58 pitches of Type1 (proper mechanics) belong to Pattern1. This means that the “expert decision” has a sensitivity of 91.4% in detecting proper mechanics. However, types 2–4 (improper mechanics) account for 37 pitches in Pattern1 and 55 in Pattern2 with a specificity of 59.8%. Although “expert decision” is good for identifying proper mechanics, it is not good enough to consistently identify improper ones. The human eye has inherent limitations and sometimes makes mistakes. For instance, trunk rotation is easier to detect with the human eye than pelvic rotation. Thus, Type3 (delayed pelvic rotation) with “on-time” trunk rotation looks similar to Type1 (proper mechanics). In addition, variation between experts is a drawback. Therefore, the new method is more precise in distinguishing between different mechanics with less variation.

Strategies for correcting improper mechanics can be determined clearly. For Type2, it is important to improve trunk flexibility. For Type3 and Type4, the strength and power of the pelvis/hips and core must be enhanced. Moreover, it is necessary to train for proper placement and angle of the leading foot and knee. Additionally, for Type4, turning-back of the trunk just before FC should be avoided.

### 4.4. Limitations

This study carries some limitations. We focused on the rotational kinematics of the pelvis and trunk. However, linear kinematics, which might also affect ball speed, were neglected. Confounders in other rotational axes of the pelvis and trunk or in other parts of the body, such as the lower and upper extremities, which might relate to pelvis and trunk rotation, could also affect ball speed and were not studied here. Small sample size is also a weakness. Further studies to improve these limitations will be conducted in the future. In addition to ball speed, the studies of kinetic effects and special programs for practical applications will also be carried out at a later point in time.

## 5. Conclusions

In accordance with the results and characteristics identified in this study, a new quantitative method with the use of “PAoFC” and “TPSoFC”, instead of “expert decision”, was developed. Pelvic and trunk rotational pitching mechanics can be classified into four types. Type1 (proper mechanics) yields significantly higher ball speeds than the other three types and is thought to feature adequate energy transfer, from the stride foot to the throwing upper limb. Types 2–4 are regarded as “improper mechanics” that result in slower ball speeds and less efficient energy transfer. The qualitative approach based on “expert decision” can identify proper pelvis and trunk rotational mechanics. However, quantitative analysis is more precise in identifying three improper types of pelvis and trunk rotational mechanics.

Furthermore, special programs, such as core strengthening and flexibility training, can be adapted to address different improper types in order to improve pitching mechanics. We hope that this new method of evaluation can help coaches, pitchers and sports scientists recognize and improve on pitching mechanics and performance.

## Figures and Tables

**Table 1:** Demographics of two groups.

Parameter	Pattern1	Pattern2	*p* Value
Hip-to-shoulder separation	Closed	Open	--
Participants	18	12	--
Age (years)	23 ± 2	24 ± 3	0.070
Body height (cm)	178 ± 4	182 ± 6	0.149
Body mass (kg)	81 ± 8	84 ± 5	0.271

Note: The data are shown in term of mean ± standard deviation.

**Table 2:** Parameters derived from the kinematic data and their abbreviations.

Abbreviation	Description
1. Parameters of timing of events	
BRt-FCt (s)	Time interval between FC and BR
MKUr (%)	Time ratio of MKU
MERr (%)	Time ratio of MER
MIRr (%)	Time ratio of MIR
2. Parameters of angle at time events	
TAoMKU (°)	Trunk angle at the moment of MKU
TAoFC (°)	Trunk angle at the moment of FC
TAoMER (°)	Trunk angle at the moment of MER
TAoBR (°)	Trunk angle at the moment of BR
TAoMIR (°)	Trunk angle at the moment of MIR
PAoMKU (°)	Pelvic angle at the moment of MKU
PAoFC (°)	Pelvic angle at the moment of FC
PAoMER (°)	Pelvic angle at the moment of MER
PAoBR (°)	Pelvic angle at the moment of BR
PAoMIR (°)	Pelvic angle at the moment of MIR
3. Parameters of trunk-pelvis separation (TPS) at time events	
TPSoMKU (°)	TPS at the moment of MKU
TPSoFC (°)	TPS at the moment of FC
TPSoMER (°)	TPS at the moment of MER
TPSoBR (°)	TPS at the moment of BR
TPSoMIR (°)	TPS at the moment of MIR
4. Parameters associated with special time events & intervals	
AC1r (%)	Time ratio of AC1
AC2r (%)	Time ratio of AC2
AC2r-AC1r (%)	Time ratio interval between AC1 and AC2
5. Parameters those are important in stride phase	
StrideL/BH (%)	Percentage of stride length normalized with body height
MKH/BH (%)	Percentage of maximal knee height normalized with body height
SFCD (°)	Stride foot contact direction

AC1 = time of first pelvic and trunk angle crossing; AC2 = time of second pelvic and trunk angle crossing; BR = ball-release; FC = foot contact; MER = maximum-shoulder-external-rotation; MIR = maximum-shoulder-internal-rotation; MKU = maximum-knee-up; TPS = trunk–pelvis separation.

**Table 3:** Results of each parameter derived from the kinematic data of two groups.

Parameter	Pattern1	Pattern2	95% CI	*p* Value
1. Parameters of timing of events				
BRt-FCt (s)	0.15 ± 0.03	0.17 ± 0.04	−0.05~0.01	0.103
MKUr (%)	−475 ± 80	−387 ± 100	−168~−8	0.032 *
MERr (%)	81 ± 5	82 ± 4	−5~4	0.717
MIRr (%)	273 ± 48	262 ± 37	−31~53	0.589
2. Parameters of angle at time events				
TAoMKU (°)	−107 ± 8	−99 ± 7	−15~−0.3	0.043 *
TAoFC (°)	−93 ± 12	−97 ± 8	−6~14	0.404
TAoMER (°)	4 ± 7	11 ± 6	−13~−1	0.019 *
TAoBR (°)	15 ± 6	22 ± 3	−12~−2	0.005 *
TAoMIR (°)	82 ± 13	95 ± 15	−25~−0.1	0.048 *
PAoMKU (°)	−128 ± 10	−133 ± 14	−6~16	0.331
PAoFC (°)	−62 ± 6	−74 ± 2	7~17	<0.001 *
PAoMER (°)	5 ± 7	−3 ± 8	0.6~14	0.035 *
PAoBR (°)	12 ± 7	4 ± 8	0.4~15	0.038 *
PAoMIR (°)	38 ± 11	37 ± 12	−9~12	0.813
3. Parameters of trunk−pelvis separation (TPS) at time events				
TPSoMKU (°)	21 ± 9	34 ± 13	−22~−3	0.011 *
TPSoFC (°)	−31 ± 8	−24 ± 6	−15~−0.5	0.037 *
TPSoMER (°)	−1 ± 7	14 ± 9	−22~−8	<0.001 *
TPSoBR (°)	3 ± 6	18 ± 9	−21~−9	<0.001 *
TPSoMIR (°)	44 ± 6	58 ± 21	−25~−3	0.017 *
4. Parameters associated with special time events & intervals				
AC1r (%)	−100 ± 29	−62 ± 11	−62~−14	0.004 *
AC2r (%)	88 ± 16	65 ± 24	6~40	0.010 *
AC2r-AC1r (%)	191 ± 27	127 ± 24	40~88	<0.001 *
5. Parameters those are important in stride phase				
StrideL/BH (%)	69 ± 4	70 ± 3	−4~3	0.651
MKH/BH (%)	65 ± 5	67 ± 6	−7~3	0.438
SFCD (°)	−7 ± 7	−10 ± 10	−5~11	0.407
6. Resultant parameter				
Ball Speed (km/h)	125 ± 6	119 ± 8	4~9	<0.001 *

Pattern1: Closed hip-to-shoulder separation; Pattern2: Open hip-to-shoulder separation; 95% CI = 95% confidence interval on the difference of the population means; MKU = maximal-knee-up; FC = foot-contact; MER = maximal-shoulder-external-rotation; BR = ball-release; MIR = maximal-shoulder-internal-rotation; TPS = trunk-pelvis separation; BRt-FCt = time interval between FC and BR; MKUr = time ratio of MKU; MERr = time ratio of MER; MIRr = time ratio of MIR; TAoMKU, TAoFC, TAoMER, TAoBR, TAoMIR = trunk angle at the moment of MKU, FC, MER, BR, MIR; PAoMKU, PAoFC, PAoMER, PAoBR, PAoMIR = pelvic angle at the moment of MKU, FC, MER, BR, MIR; TPSoMKU, TPSoFC, TPSoMER, TPSoBR, TPSoMIR = TPS at the moment of MKU, FC, MER, BR, MIR; AC1= time of 1st pelvic and trunk angle crossing; AC2 = time of 2nd pelvic and trunk angle crossing; AC1r = time ratio of AC1; AC2r = time ratio of AC2; AC2r-AC1r = time ratio interval between AC1 and AC2; StrideL/BH = percentage of stride length normalized with body height; MKH/BH = percentage of maximal knee height normalized with body height; SFCD = stride foot contact direction; * *p* value < 0.05. Note: The data are shown in term of mean ± standard deviation and 95%CI. Most of the data are rounded to the nearest whole number (except BRt-FCt).

**Table 4:** Correlation coefficients and cut-off values from ROC of each parameter correlated with ball speed and their Cohen’s *d* effect sizes (sorted by absolute value of correlation coefficient in order of high to low).

Parameter	Correlation Coefficient *	Cut-Off Value from ROC	Cohen’s *d*
PAoFC (°)	0.613	−69.95	2.68
BRt-FCt (s)	−0.527	0.19	−0.57
MKUr (%)	−0.454	−401.69	−0.97
TPSoFC (°)	−0.335	−24.92	−0.99

MKU = maximal-knee-up; FC = foot-contact; BR = ball-release; TPS = trunk–pelvis separation; PAoFC = pelvic angle at the moment of FC; BRt-FCt = time interval between FC and BR; MKUr = time ratio of MKU; TPSoFC = TPS at the moment of FC; ROC = Receiver operating characteristic; * the FDR-adjusted *p*-value < 0.05.

**Table 5:** Ball speed and pitch count percentage of different types using the new method.

			The New Method				*p* Value
			Type1	Type2	Type3	Type4	
Ball speed (km/hr)			126.1 ^a^ ±5.3	120.2 ^b^ ±9.4	120.8 ^b^ ±5.6	120.5 ^b^ ±8.9	<0.001 *
Expert’s decision		Pattern1	58.9% (53/90)	10% (9/90)	12.2% (11/90)	18.9% (17/90)	--
		Pattern2	8.3% (5/60)	23.3% (14/60)	31.7% (19/60)	36.7% (22/60)	--

^a,b^ the same superscripts indicate that no statistically significant difference are between the indicated groups (*p* > 0.05); * *p* value < 0.05. Note: Analysis of variance (ANOVA) is used for analysis of ball speed, and the results are shown in term of mean ± standard deviation.

## Data Availability

The data are not publicly available due to privacy and ethical issues.
